# Lgr4 in Ocular Development and Glaucoma

**DOI:** 10.1155/2013/987494

**Published:** 2013-06-05

**Authors:** Stefan Siwko, Li Lai, Jinsheng Weng, Mingyao Liu

**Affiliations:** ^1^Center for Cancer and Stem Cell Biology, Institute for Biosciences and Technology, Texas A & M Health Science Center, Houston, TX 77030, USA; ^2^Shanghai Key Laboratory of Regulatory Biology, Institute of Biomedical Sciences and School of Life Sciences, East China Normal University, 500 Dongchuan Road, Shanghai 200241, China; ^3^Department of Lymphoma and Myeloma, MD Anderson Cancer Center, 1515 Holcombe Boulevard, Houston, TX 77030, USA

## Abstract

The leucine-rich repeat-containing G protein-coupled receptor 4 (LGR4, also called GPR48) plays a key role in multiple developmental processes, and mice lacking *Lgr4* display anterior segment dysgenesis leading to early-onset glaucomatous retinal ganglion cell loss as well as defective eyelid formation. This paper will review Lgr4 signaling and its regulation of the Axenfeld-Rieger syndrome gene *Pitx2*, a crucial developmental transcription factor. In addition, Wnt signaling plays an important role in eye development, with Norrin functioning to activate the Wnt receptor Frizzled 4 required for proper retinal vascularization. Recent discoveries identifying Lgr4 as a receptor for Norrin highlight the potential for Lgr4 function in retinal vascularization. Finally, several unanswered questions impeding a full understanding of Lgr4 in glaucoma are considered as avenues for further research.

## 1. Introduction

Glaucoma is the second leading cause of blindness worldwide, but its etiology is complex and only partially understood. Frequently associated with elevated intraocular pressure (IOP) leading to a stereotypical pattern of retinal ganglion loss and cup excavation, glaucoma may result from closure of the iridocorneal angle, blockage of the trabecular meshwork responsible for aqueous humor outflow, pupil block of the space between the lens and iris necessary for humor circulation between the anterior and posterior chambers, and several other causes. Loss of the leucine-rich repeat domain-containing G protein-coupled receptor 4 (Lgr4, also called Gpr48) has been implicated in anterior segment dysgenesis including iridocorneal attachment and elevated intraocular pressure leading to early onset retinal ganglion cell loss in mice that is similar to glaucomatous damage in humans [1]. Recently published work identifies Lgr4 as a receptor for Norrin, a secreted protein with established roles in retinal neuron protection and retinal vascularization and therefore suggests an additional mechanism by which Lgr4 functions to prevent glaucoma [2]. This paper will provide an overview of Lgr4 signal transduction and its role in a wide variety of developmental processes, followed by a focus on recent developments in the role of Lgr4 in glaucoma. 

## 2. Lgr4 Signaling

The leucine-rich repeat domain-containing G protein-coupled receptors (LGRs) feature a large N-terminal extracellular domain containing multiple leucine-rich repeats and are subdivided into three groups. One group consists of the three glycoprotein hormone receptors: lutenizing hormone receptor, follicle-stimulating hormone receptor, and thyroid stimulating hormone receptor. The second group contains LGR4–6, three receptors sharing high homology (~50% sequence identity) that were recently found to act as receptors for the Wnt-potentiating R-spondins, indicating that this group can signal through both G protein-coupled as well as Wnt-signaling pathways. The third group is comprised of the relaxin receptors LGR7 and 8.

**Figure 1:**
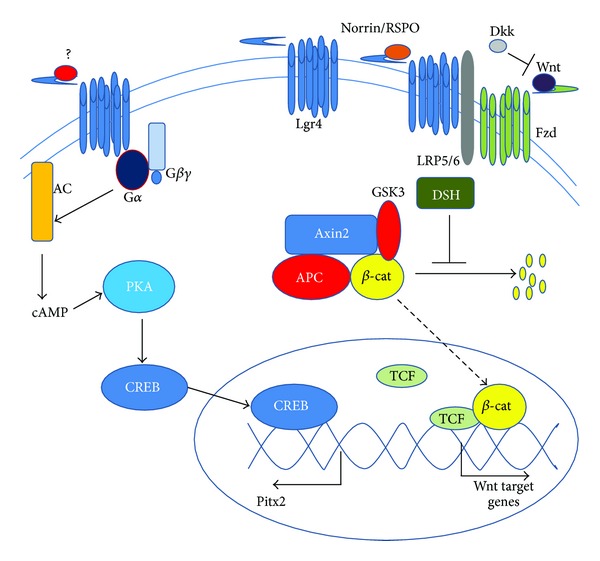
Signaling pathways downstream of Lgr4. Left: binding of unknown ligands (?) leads to G_*α*s_ activation of adenylyl cyclase (AC) and resulting increase in intracellular cyclic AMP levels (cAMP). Elevated cAMP activates protein kinase A (PKA), which phosphorylates CREB inducing its nuclear translocation and regulation of CRE target genes such as *Pitx2*. Right: Norrin or R-spondin (Norrin/RSPO) binding to Lgr4 augments the response of Frizzled (Fzd) and LRP5/6 to Wnt binding, leading to activation of Disheveled (DSH). DSH blocks the GSK3/Axin2/APC-mediated degradation of *β*-catenin (*β*-cat), allowing *β*-catenin accumulation and nuclear translocation (dashed arrow) where *β*-cat binds TCF/LEF (TCF) family transcription factors to regulate Wnt target gene expression. Dikkopf (Dkk) is a competitive inhibitor of Wnt binding to Fzd.

Lgr4 has been shown to signal through classical G_*α*s_-mediated signaling in multiple systems. In this pathway (Figure 1), ligand binding to Lgr4 leads to G-protein activation through GTP binding. Coupled G_*α*s_ then dissociates from Lgr4 to activate adenylyl cyclase resulting in elevated levels of the second messenger cyclic AMP (cAMP); cAMP, in turn, activates protein kinase A (PKA), which phosphorylates the transcription factor Cre-binding protein, leading to elevated expression of target genes containing CRE binding motifs in their promoter. Known Lgr4 targets regulated through cAMP/PKA/CRE signaling include the mineralocorticoid receptor [3], estrogen receptor *α* in the male reproductive tract [4], ATF4 in bone development and definitive erythropoiesis [5, 6], and Pitx2 in eye development [1]. Significantly, the ligand(s) initiating cAMP/PKA signaling by Lgr4–6 remains unidentified. However, a constitutively active mutant, T755I Lgr4, has been reported which results in elevated cellular cAMP levels and CREB activity [1, 7].

The Lgr4 family members Lgr5 and 6 have been implicated in stem cell maintenance in a variety of tissues. Lgr5 is a stem cell marker in the small intestine [8], stomach [9], liver [10], hair follicle [11], and, most recently, mammary gland [12, 13]. Lgr6 marks the most primitive kind of epidermal stem cell [14]. A single Lgr5 expressing colon cell was able to generate organoids that persist in culture and can engraft to form functional crypts* in vivo* [15]. Conditional loss of *Lgr5* leads to depletion of stem cells in the mammary gland [12], implying that Lgr5 signaling has a functional role in stem cell self-renewal. The most likely mechanism for this role is by a second signal transduction pathway: Lgr mediation of Wnt signaling potentiation by R-spondin. R-spondin binding to LGR4–6 inhibits ZNRF3 and RNF43, negative regulators of Wnt signaling which promote degradation of the Wnt receptor Frz and the Wnt coreceptors LRP5/6 [16]. Thus, Lgr4 and its family members function to increase the membrane concentration of Wnt receptors in the presence of R-spondin, enhancing the signaling response to low levels of Wnt ligand. An alternative mechanism for R-spondin signaling has also been proposed, in which R-spondin-bound Lgrs bind directly to LRP6 to augment LRP6 phosphorylation in response to Wnt-Fzd binding [17]. Clathrin was also reported to be required for Lgr4 mediation of R-spondin *in vitro* [18]. Most recently, Lgr4 was shown to be a receptor for Norrin, another canonical Wnt signaling potentiator [2], providing another means by which Lgr4 modulates Wnt signaling.

## 3. Lgr4 in Development

Lgr4 mRNA expression in mice was first detected at E7 and in adult mice was the highest in liver, then kidney, with moderate expression in muscle, heart, and brain, and low levels in testes and lung [19]. Mazerbourg et al. (2004) [20] first described the mouse expression pattern of Lgr4 protein, using both IHC staining of wild-type tissue as well as transgenic mice expressing β-galactosidase from the Lgr4 promoter. They noted moderate Lgr4 expression in neonatal kidney, adrenal, stomach, spine, ribs, brain, nasal cavity, heart, and intestines, with lower levels in liver, lung, and spleen. No Lgr4 was detected in skeletal muscle or pancreas. Adults had a very similar pattern of Lgr4 expression, with reduced heart Lgr4 and higher liver levels; also, no lung or spleen expression was detected in adult *Lgr4* ^+/−^ [20]. Lgr4 mRNA expression in adult humans is the highest in the pancreas, with moderate levels in liver, heart, and muscle, and very low brain and kidney expression [19]. 

Two approaches have been used by different labs to generate *Lgr4* ^−/−^ mice. In one approach, a gene trap cassette was inserted in the first intron to generate a chimeric mRNA containing the N-terminal Leucine-rich repeat of Lgr4 fused to the CD4 transmembrane domain and the β-galactosidase coding sequence [1, 20]. This approach showed that Lgr4 is an essential gene for embryonic development; 60% of expected *Lgr4* ^−/−^ pups died in utero, and the majority of remaining Lgr4 null pups died perinatally in a C57Bl6J X Swiss Webster background; modest (~10%) declines in expected numbers of *Lgr4* ^+/−^ mice were also reported [20]. *Lgr4* ^−/−^ pups showed embryonic growth retardation (14% decrease in *Lgr4* ^−/−^ neonatal pup weight), with a significant decrease in liver and kidney weight [20]. Background strain has a strong effect on the embryonic and perinatal survival of *Lgr4* ^−/−^ mice, with higher lethality in 129Ola x C57Bl6 mice [21], but 60% of Lgr4 null mice on a CBA background survive [22]; most CD1 strain *Lgr4* ^−/−^ mice also survive to adulthood [23]. The alternative approach is to perform targeted knockout of Lgr4 exon 18, containing the seven-transmembrane domain. This approach revealed a similarly high level of embryonic lethality, with embryonic kidney hypoplasia [21], later shown to be accompanied by premature ureteric bud differentiation [24]. 

A wide variety of developmental defects have been reported in *Lgr4* ^−/−^ mice. In addition to the embryonic lethality and decreased growth noted above and the eye defects discussed below, midgestational erythropoiesis is disrupted in *Lgr4* ^−/−^ embryos, with a 32% decrease in Ter119^+^ cells at E13.5 as compared to wild type [6]. Lgr4 loss delays osteoblast differentiation, resulting in decreased embryonic bone formation; bone formation kinetics and bone mineral density were decreased through adulthood in *Lgr4* ^−/−^ mice [5]. *Lgr4* ^−/−^ mice also lack a gall bladder and cystic duct, although the common hepatic duct and intrahepatic bile duct appeared unchanged [25]. Lgr4 regulates expression of the mineralocorticoid receptor, implicating Lgr4 in electrolyte homeostasis [3]. Both male [4, 22, 23, 26] and female [27] reproductive tract formation is impaired in *Lgr4* ^−/−^, leading to infertility in homozygous null mice. Intestinal epithelial cell proliferation was reduced in *Lgr4* ^−/−^ mice, with an 80% decrease in crypt Paneth cells, suggesting that Lgr4 plays a crucial role in intestinal stem cell maintenance [28]; Lgr4 also appears to play a protective role against inflammatory bowel disease [29]. 

Conditional knockout approaches have also been used to avoid the embryonic lethality of Lgr4 loss and have revealed additional developmental phenotypes. Mice with specific ablation of Lgr4 in cells expressing keratin 5 showed focal alopecia and fewer hair placodes, implicating Lgr4 action in hair follicle development [30]. Loss of Lgr4 in keratin 5^+^ cells also disrupted mammary gland branching morphogenesis and delayed ductal elongation [31] and was proposed to impair oviduct function in promoting embryo development [27]. Therefore, Lgr4 is a key regulator of organ development in a wide variety of extraocular systems. 

### 3.1. Lgr4 in Eye Development

**Figure 2:**
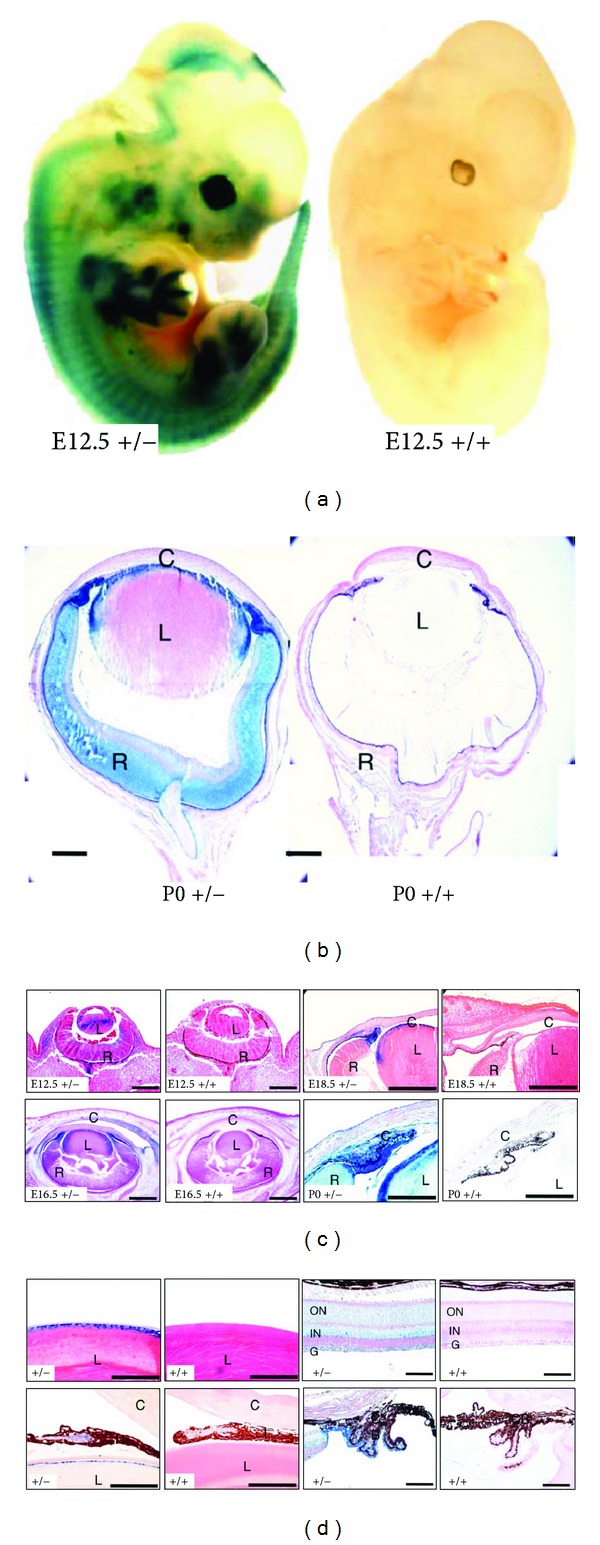
Temporal and spatial expression of Lgr4 in mouse eyes. (a) Expression of Lgr4 at embryonic day E12.5 using *β*-galactosidase staining in Lgr4 heterozygous (+/−) and wild-type control (+/+) mice. (b) Expression of Lgr4 at postnatal day 0 (P0). Scale bar = 170 *μ*m. (c) Temporal expression of Lgr4 during different stages of anterior segment development. At E12.5 days, Lgr4-expressing mesenchymal cells are located between the surface ectoderm and the lens and the inner layer cells of the optic cup. At E16.5–E18.5, Lgr4 expression is high at the tips of the optic cup and in the surrounding mesenchymal tissue. In newborn embryos, Lgr4 is highly expressed in the iris stroma, ciliary body, corneal epithelium, keratocytes, and endoepithelial cells. Wild-type (+/+) mice lack expression of *β*-galactosidase. Scale bars = 170 *μ*m. (d). Expression of Lgr4 in adult mouse tissue. Staining was found in lens epithelial cells, retinal ganglion cells, inner nuclear layer, iris stroma, and the outer layer of the ciliary body. C: cornea; I: iris; R: retina; L: lens; CB: ciliary body; G: ganglion cells; IN: inner nuclear layer; ON: outer nuclear layer. Scale bars = 85 *μ*m. Originally published PNAS 105(16): 6081-6. Copyright 2008 National Academy of Sciences USA.

Lgr4 is normally expressed in a finely tuned spatiotemporal pattern in the developing eye. Using Lgr4-driven β-galactosidase expression to probe for Lgr4 expression in Lgr4 heterozygotes, we found that at E12.5, Lgr4 is primarily expressed in a layer of mesenchymal cells between the surface ectoderm and the optic cup; limited expression is also detectable in the lens and the outer layer of the optic cup (Figure 2). By E16.5, high-level Lgr4 expression is seen in the tips of the optic cup and surrounding mesenchyme, which later form the iris and ciliary body, with lower levels in the cornea, retina, and lens. Neonatal mice retain strong expression in the ciliary body, iris stroma, lens, and corneal epithelial cell layer, with lower expression in keratocytes and endoepithelial cells. Adult *Lgr4* ^+/−^ mice express Lgr4 in the lens epithelium, retinal ganglion and inner nuclear layer, iris stroma, and outer ciliary body cell layer [1]. 

Using the gene trap approach described above, we generated *Lgr4* ^−/−^ mice which had multiple ocular defects. 25 out of 47 *Lgr4* ^−/−^ mice had microphthalmia, with a much higher incidence in males (75%) compared to females (37%), suggesting that Lgr4 interacts with a sex-linked factor in this phenotype [1]. Partial corneal opacity, usually accompanied by corneal neovascularization, was present in 18 of 47 mice. A variety of keratopathies (corneal epithelial plug, corneal inflammation, corneal cyst-like structures, and corneal vascular pannus) were each found in 4 or more mice, with a higher frequency in male mice [1]. Cataracts formed in 13 of 47 *Lgr4* ^−/−^ mice, which were correlated with higher levels of insoluble *α*A-crystallin in the lenses of *Lgr4* ^−/−^ mice.

Furthermore, Lgr4 plays an essential role in eyelid development. 85% of *Lgr4* ^−/−^ mice had a failure of eyelid fusion in utero leading to eye open at birth, and adult *Lgr4* ^−/−^ mice exhibited exposure keratitis [32]. In a separate paper using an Lgr4 exon 18 deletion, *Lgr4* ^−/−^ mice had complete eye open at birth penetrance, but these mice did not survive to adulthood [33]. Lgr4 is normally expressed in the eyelid tip basal epithelium and mesenchyme at E14–E16. *Lgr4* ^−/−^ mouse eyelids were morphologically similar to those of *Lgr4* ^+/+^ at E12.5 but had decreased eyelid epithelial cell proliferation. Eyelid extension towards the corneal center was noticeably decreased by E15.5 in the absence of Lgr4 [33], with fewer filopodia [32]. Cultured *Lgr4* ^−/−^ keratinocytes had decreased migration [33] and proliferation and decreased phospho-EGFR, suggesting that Lgr4 regulates EGFR activation [32]. Inhibition of EGFR reduced keratinocyte proliferation and migration to levels seen in *Lgr4* ^−/−^ cells, and inhibitory antibodies against HB-EGF also reduced wild-type keratinocyte proliferation to *Lgr4* ^−/−^ levels, suggesting that Lgr4 may activate EGFR signaling in keratinocytes through upregulation of metalloproteases that generate HB-EGF; however, this model awaits confirmation* in vivo* [34]. 

Finally, anterior segment dysgenesis (ASD) was common in mice lacking Lgr4. *Lgr4* ^−/−^ mice displayed iris hypoplasia with decreased stroma and smooth muscle as early as postnatal day 4, diminished ciliary body size and folding, compressed trabecular meshworks with fewer and smaller beams, and a sharper iridocorneal angle that in some cases (13%) was completely closed. *Lgr4* ^−/−^ retinas had detectable loss of inner nuclear layer ganglion cells and disruption of the outer nuclear layer beginning at 6 months of age in 42% of mice examined (10 of 24), strongly implicating ASD resulting from Lgr4 loss in early-onset glaucoma [1]. Several lines of evidence implicate the Axenfeld-Rieger syndrome-related gene *Pitx2* as a key mediator of Lgr4 in eye development. First, the ASD phenotype in *Lgr4* ^−/−^ mice closely matches that seen in *Pitx2* ^+/−^ mice, including iris hypoplasia and periocular musculature defects. Second, Pitx2 was the only transcription factor out of a panel of fourteen key eye development genes to be downregulated in *Lgr4* ^−/−^ mice. Finally, Pitx2 was shown to be a direct downstream target of Lgr4 signaling through the cAMP/PKA/CREB pathway [1]. 

## 4. Pitx2 and Axenfeld-Rieger Syndrome

Axenfeld-Rieger syndrome (ARS) is a rare genetic disease generally with autosomal dominant inheritance characterized by ocular disorders (potentially including iris hypoplasia, corectopia, pseudopolycoria, posterior embryotoxon, and iris strands connecting to the trabecular meshwork or other angle structure anomalies) resulting in elevated intraocular pressure, sometimes accompanied by craniofacial abnormalities (telecanthus, hypertelorism) or dental defects (small or missing teeth). ARS patients have a high risk for glaucoma [35]. Cardiovascular abnormalities are also reported in ARS patients [36–39], as well as hearing loss in some cases [36, 40]. Mutations in either *PITX2* or *FOXC1* have been estimated to account for 40% of ARS cases [35, 41–45]. Pitx2 is a paired-like homeodomain transcription factor. Mice heterozygous for *Pitx2* display multiple anterior segment defects similar to ARS, including corneal endoderm and iris stroma agenesis, corneal mesothelial thickening, coloboma formation, and shortened ventral retina, and *Pitx2* ^−/−^ mice are embryonic lethal due to incomplete closure of the ventral body wall [46, 47]. Loss of extraocular musculature has also been reported in *Pitx2* heterozygotes, with a more severe phenotype in *Pitx2* ^−/−^ mice [48]. Pitx2 is normally expressed in the neural crest during development beginning at E9.5, as well as in the developing eye mesoderm, hyaloid space, and eyelid mesenchyme [49, 50]. There is a strong dosage dependence of Pitx2 for proper development; transgenic mice overexpressing PITX2A in the corneal mesenchyme and iris show corneal hypertrophy, corneal opacification, iridocorneal attachments, and retinal degeneration [51]. Intriguingly, a downstream target of Pitx2 is the Wnt-signaling antagonist *Dkk2*, which plays a crucial role in regulating anterior segment morphogenesis [52]. 

## 5. Norrin and Wnt Signaling in Eye Development

Canonical Wnt/β-catenin signaling plays an essential role in development and in tissue homeostasis. Key components of the pathway are the Wnt proteins, a family of 19 secreted glycoproteins, the Frizzled (Fzd) receptors, which are seven membrane-spanning receptors for Wnt, and the Wnt coreceptors LRP5 and 6. Wnt binding results in activation of the downstream transducer Dishevelled (Dsh), which phosphorylates glycogen synthase kinase 3 (GSK-3) resulting in inactivation of the Axin/APC complex that normally ubiquitylates β-catenin (Figure 1). As a consequence, β-catenin accumulates and translocates to the nucleus, where it associates with Tcf/Lef transcription factors to regulate gene transcription. Negative regulators of Wnt signaling include the Dikkopf (Dkk) and secreted Frizzled-related proteins (sFRP) which bind to Frz or Wnt, respectively, to inhibit cascade activation. Loss of β-catenin in the periocular ectoderm results in ectopic lentoid body formation whereas presumptive lens ectoderm expression of dominant active β-catenin suppresses lens development [53], suggesting that low levels of β-catenin signaling are required for proper lens formation. Ectoderm-specific active β-catenin expression disrupts optic cup patterning resulting in a lack of cornea, conjunctiva, and eyelid development [54]. *Dkk2* ^−/−^ mice display a transformation of corneal epithelium into a stratified epithelium [55], iridocorneal adhesion, eye open at birth phenotype with hypomorphic eyelids and ectopic hair follicles in the presumptive conjunctiva, and ectopic corneal stroma vasculature [52]. Therefore, proper spatiotemporal control of Wnt/β-catenin pathway signaling intensity is essential for anterior segment development.

In addition to playing a crucial role in anterior segment development, Wnt signaling was reported to have a significant ongoing function in trabecular meshwork (TM) cell regulation. RNA differential display of cultured human TM cells identified elevated expression of the Wnt antagonist secreted Frizzled-related protein 1 (sFRP1) in cells from glaucomatous donors, and perfusion of sFRP1 into cultured human eyes resulted in decreased canonical Wnt signaling and reduced aqueous outflow. Intraocular injection of adenoviral vectors to express sFRP-1 in BALB/c mice resulted in a twofold increase in intraocular pressure (IOP), an increase that was partially reversible through topical application of a GSK-3 inhibitor, suggesting that sFRP-1 increased IOP through inhibiting canonical Wnt/β-catenin signaling [56]. 

Intriguingly, Pitx2 had been reported to be a direct Wnt target gene in culture [57, 58], and *Dkk2* ^−/−^ mice have elevated Pitx2 expression in the corneal stroma, suggesting the existence of a negative feedback loop regulating Pitx2 expression during eye development [52]. We speculate that Lgr4 may play a crucial role in integrating these signaling circuits, as Lgr4 binding to R-spondin potentiates Wnt signaling, leading to Pitx2 expression, and activation of G protein signaling through a binding of an unknown ligand to Lgr4 results in Pitx2 activation and resulting Dkk2 expression, which then inhibits Wnt signaling even in the presence of both R-spondin and Wnt. Whether the failure of this signaling circuit is responsible for the ocular defects in *Lgr4* ^−/−^ mice remains to be determined. 

### 5.1. Norrin in Retinal Vascularization and Function

Norrin has been shown to enhance Wnt signaling *in vitro* and to selectively bind to the Wnt receptor Fzd4 with high affinity to activate canonical Wnt/β-catenin signaling [59]. Mutations in the *NDP* gene encoding Norrin result in Norrie disease, an X-linked congenital syndrome characterized by retinal vascularization failure leading to blindness, often accompanied by microcephaly, deafness, hypogonadism, or mental retardation. Familial exudative vitreoretinopathy, a less severe disruption in peripheral retina vascularization, can also be caused by mutations in *NDP,* or alternatively by mutations in *Fzd4* or *LRP5*. Loss of the mouse homologue, *Ndp*, causes defects in retinal vasculature which lead to blindness as well as cochlear vasculature, and results in female infertility due to defects in decidualization [59–63]. Curiously, a similar defect in retinal vascularization has been reported in mice lacking the Wnt receptor Fzd4 [59] or coreceptor Lrp5 [64]. Norrin has very recently been reported to be a ligand for Lgr4–6, suggesting that it plays a role in Wnt signal potentiation similar to that played by R-spondin family members [2]. Norrin is normally expressed by Müller glial cells of the mouse retina [65]; however, retinal vascularization defects in *Ndp* ^y/−^ mice are overcome by lens-specific expression of Norrin [66], implying a paracrine mode of action that does not require spatial concentration gradient formation. Systemic Norrin overexpression is embryonic lethal, marked by defective angiogenesis, but can be rescued on either an *Fzd4* ^−/−^ or an *Lrp5* ^−/−^ background [65]. Retinal pigment epithelial cell expression of Norrin ablated the extent of oxygen-induced retinopathy in young (P7–11) mice, implying a protective effect of Norrin on retinal vasculature [67]. Curiously, inhibition of Fz4 signaling reduced the recovery from oxygen-induced retinopathy [68]. Angiopoetin-2, an angiogenic growth factor expressed in the retinal microvasculature endothelium, has been proposed to be a downstream mediator of Norrin's role in angiogenesis, based on (1) a similar defect in retinal vascularization in *Ang-2* ^−/−^ mice, (2) evidence for Norrin regulation of Ang-2 expression *in vivo*, and (3) loss of Norrin-induced increased endothelial cell proliferation in the presence of Ang-2-blocking antibodies [67, 69, 70].

In addition to the retinal vascularization defects, *Ndp* ^y/−^ mice exhibit a loss of retinal ganglion cells (RGCs), with concomitant decrease in normal retinal function [60, 61, 66]. Lens-specific transgenic expression of Norrin increased RGC proliferation and resulted in thicker retinas in mice two days postnatal [66]. Norrin has also been reported to have a neuroprotective effect on retinal ganglion cells. Ocular injection of Norrin reduced the extent of RGC apoptosis and axon loss following NMDA injection [71], which is in agreement with a report demonstrating antiapoptotic effects of Norrin on a cultured RGC cell line [72]. The RGC protective effect of Norrin was partially mediated through induction of several growth factors (including fibroblast growth factor 2, ciliary neurotrophic growth factor, and brain-derived neurotrophic growth factor) by Müller cells, and partly through a direct effect of Norrin on RGCs [71]. Intriguingly, no activation of canonical Wnt/β-catenin signaling was detected in RGCs following Norrin treatment, suggesting that Norrin acts through a yet-undiscovered signal transduction pathway to prevent excitotoxicity in RGCs. Given the recent finding that Lgr4 is able to bind Norrin [2], this leads to the speculation that this neuroprotective effect of Norrin may be mediated by G protein-coupled signaling downstream of Lgr4.

## 6. Future Directions

In summary, there is strong evidence that Lgr4 is a key regulator of Pitx2 in anterior segment formation and that disruption of this signaling network can result in early-onset glaucoma in a mouse model. Furthermore, tantalizing new evidence suggests that Lgr4 functioning as a receptor for Norrin may be implicated in retinal vascularization and/or other functions of Norrin, such as retinal neuron protection from damage. However, a host of critical questions remain. First, what is the endogenous ligand activating Lgr4 signaling through the cAMP/PKA pathway resulting in regulation of Pitx2 and multiple other downstream targets? Identification of this unknown Lgr4 activator is essential for mapping the spatiotemporal profile of Lgr4 activity during development and may serve as a starting point for designing small-molecule agonists or inhibitors for potential therapeutic use. Second, which Norrin functions are mediated by Lgr4 or its close family members? Could Norrin be the unknown sex-linked factor responsible for the higher incidence of microphthalmia in male *Lgr4* ^−/−^ mice? Deciphering the relative importance of classical G protein-coupled versus Wnt/β-catenin signaling in Lgr4 function remains to be unraveled.

A third unresolved question concerns additional functions of Lgr4. An area of particular therapeutic interest is the use of stem cells for glaucoma treatment. Some evidence exists suggesting the existence of stem or progenitor cells in the region of Schwalbe's line (called by several names including Schwalbe's line cells, trabecular meshwork insert cells, and progenitors for endothelium and trabeculum, and functionally assayed using sphere formation *in vitro*; see [73] for an excellent review). Given the roles of Lgr5 and Lgr6 in stem cell self-renewal in a wide variety of tissues, together with the established function of R-spondin (and Norrin) to potentiate Wnt signaling through Lgr4–6, we speculate that Lgr4 or its family members may play a key role in maintenance of stem/progenitor cells in this region. Understanding such a role will be vital in progress towards using stem cells to regenerate a blocked trabecular meshwork as an approach towards relieving intraocular pressure.
